# New buffer systems for photopainting of single biomolecules†

**DOI:** 10.1039/d3lf00125c

**Published:** 2023-10-07

**Authors:** Christoph Naderer, Heinrich Krobath, Dmitry Sivun, Georgii Gvindzhiliia, Thomas A. Klar, Jaroslaw Jacak

**Affiliations:** a School of Medical Engineering and Applied Social Sciences, University of Applied Sciences Upper Austria Garnisonstraße 21 4020 Linz Austria jaroslaw.jacak@fh-linz.at; b Institute of Theoretical Physics, Johannes Kepler University Linz Altenberger Straße 69 4040 Linz Austria; c Institute of Applied Physics, Johannes Kepler University Linz Altenberger Straße 69 4040 Linz Austria

## Abstract

We present newly developed buffer systems that significantly improve the efficiency of a photochemically induced surface modification at the single molecule level. Buffers with paramagnetic cations and radical oxygen promoting species facilitate laser-assisted protein adsorption by photobleaching (LAPAP) of single fluorescently labelled oligonucleotides or biotin onto multi-photon-lithography-structured 2D and 3D acrylate scaffolds. Single molecule fluorescence microscopy has been used to quantify photopainting efficiency. We identify specific cation interaction sites for members of the cyanine, coumarin and rhodamine classes of fluorophores using quantum mechanical calculations. We show that our buffer systems provide an up to three-fold LAPAP-efficiency increase for the cyanine fluorophore, while keeping excitation parameters constant.

## Abstract 2

We present novel buffer systems with paramagnetic cations and radical oxygen promoters which significantly enhance single-molecule LAPAP-efficiency due to specific cation-fluorophore interactions.

## Graphical abstract

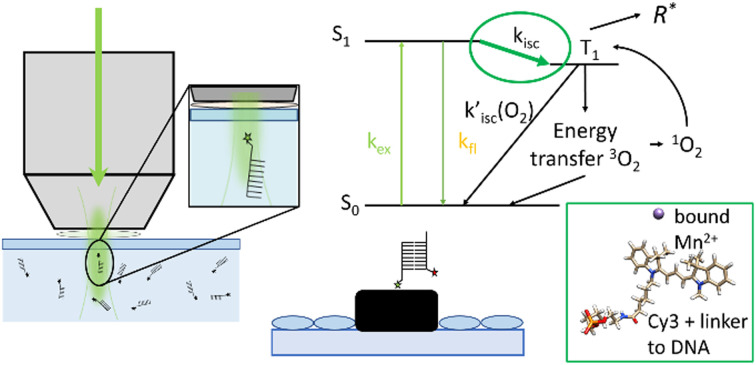

## 1 Introduction

Photopatterning techniques are attracting increasing interest due to their widespread applications in lithography, biointerfaces, and microfluidics.^1–3^ Manipulation of surface functionalities is important for applications involving adaptive surface properties.^4–14^ Surfaces that can regulate biological functions by stimuli-responsive compounds allow the control of molecular uptake, manipulation of cell adhesion and sensing,^12,15–26^ or the induction of protein denaturation.^27,28^ Physical and chemical properties can be (ir-)reversibly tuned under a stimulus such as light, temperature, chemicals, pH, or an electric field.^29–40^ Light provides high spatio-temporal resolution to control the functions of such surfaces without a direct contact. This is achieved by photoisomerization, -coupling or -cleavage through chemical bond breakage/formation,^20,41–48^ or addition of photo-switchable molecules such as azobenzene,^49–55^ spiropyran,^56–58^ dithienylethene,^59,60^ and molecular shuttles.^61–63^ Such photo-switchable modifications can be switched between multiple molecular states *via* light irradiation.^64–69^

There is a continuing need for biologically active molecular concentration gradients or local, precise surface modifications to decipher cellular functions *in vitro*.^70–72^ One possibility to improve the modification quality of the surface is irreversible patterning *via* laser-assisted protein adsorption by photobleaching (LAPAP).^73^ Here, photopainting is introduced by intentional bleaching of fluorophores, where the formed radicals subsequently bind to the surface. LAPAP has been introduced using lamps and photomasks to covalently bind biotin-4-fluorescein (B4F) to bovine serum albumin (BSA) adsorbed on a glass surface for surface functionalization.^74^ These experiments yielded resolution-limited molecular patterns. Recently, the resolution has been extended to the nanoscale by combining LAPAP and nanostructured polymeric anchors fabricated *via* stimulated emission depletion (STED) nanolithography.^75,76^

Our motivation to improve the LAPAP efficiency was driven by spectroscopic studies by Stennett *et al.* and Ciuba *et al.*^77,78^ It has been demonstrated that for TAMRA, Cy3/Cy3B and Cy5 in aqueous solvent, Mn^2+^ in the solution increases the intersystem crossing (ISC) efficiency. Related studies on the applicability of cyanine dyes show that they reversibly switch between a fluorescent state and a dark state when illuminated at different wavelengths.^79,80^

In the context of LAPAP, photobleaching and radical formation can be induced using paramagnetic transition metal- (*e.g.* Zn^2+^) or heavy metal ions (*e.g.* iodine) as well as ROS-promotors.^81–83^ The role of ROS in the photobleaching process of several classes of fluorophores fostered the idea of using photoactive molecules as ROS promoters for increased photopainting efficiency.^84^ Water soluble porphyrin is a well-known ROS promoter, due to singlet oxygen formation.^85–88^

In this work, we present new buffer systems for improved LAPAP patterning efficiency. We characterise the effect of Mn^2+^ as well as ruthenium porphyrin (RuPo) on three fluorophore class representatives by optimizing buffer systems for single-molecule LAPAP.

Our buffer systems enhance the efficiency for single molecule LAPAP (smLAPAP) on Multi-Photon Lithography (MPL) structured scaffolds. Experiments with representatives of cyanine- (Cy3), coumarin- (ATTO 390) and rhodamine- (ATTO 514) fluorophores have been performed. The interaction of Mn^2+^ has been corroborated by quantum mechanical calculations yielding optimal fluorophore/Mn^2+^ complexes with specific ion binding sites. Using our optimized buffers, single oligonucleotides and biotins have been photopainted efficiently on 2D/3D acrylate scaffolds with low unspecific surface adsorption. Photopainting efficiency has been confirmed using single molecule fluorescence microscopy (SMFM).

## 2 Experimental section

### 2.1 Materials and methods

#### 2.1.1 Structuring and photoresist

For multi-photon lithography (MPL), a mixture of the monomers Ormocomp^89,90^ (Micro Resist Technology, Germany) and poly(ethylene glycol) diacrylate (PEGDA, *M*_n_ = 575, Sigma Aldrich, USA) was used in a 4 : 1 mass ratio. 1 wt% of Irgacure 819 (Sigma Aldrich, USA) was added as photoinitiator.

#### 2.1.2 Chemicals

Bovine serum albumin (BSA, Sigma Aldrich) was used for surface passivation. LAPAP was performed with Cy3 conjugated to the model oligonucleotide NP77 (ref. 91) (sequence: CGC TAG ACT AGT ACA ATT GGC AGA CTG CCT TGA TCG GTA CGA ACA GAT CGC GTC AGG TAT, 3′ modification: C6-spacer amino modification – Cy3 carboxylic acid) (Cy3-NP77, Microsynth AG, Switzerland) (“Cy-LAPAP”), ATTO 390 conjugated to biotin (ATTO390-biotin, ATTO-TEC GmbH, Germany) (“Cou-LAPAP”) and ATTO 514 conjugated to biotin (ATTO514-biotin, ATTO-TEC GmbH, Germany) (“Rho-LAPAP”). For visualization of the fluorophore–biomolecule conjugates bound *via* LAPAP, ATTO 647N conjugated to the complementary oligonucleotide cNP77 (sequence: ATA CCT GAC GCG ATC TGT TCG TAC CGA TCA AGG CAG TCT GCC AAT TGT ACT AGT CTA GCG, 5′ modification: C6-spacer amino modification – ATTO 647N) (ATTO647N-cNP77, Microsynth AG, Switzerland) was used for Cy-LAPAP and ATTO 655 conjugated to streptavidin (ATTO655-SA, ATTO-TEC GmbH, Germany) was used for Cou-LAPAP and Rho-LAPAP. Ethylenediamine tetra acetic acid disodium salt (EDTA) (Roth, Germany) dissolved in double-distilled water and phosphate buffered saline (PBS) were used for washing the sample.

#### 2.1.3 Buffer systems

Buffer I consisted of double-distilled water (Labochem, Germany) (Cy-LAPAP) or PBS (Cou-LAPAP, Rho-LAPAP). Buffer II consisted of buffer I with 2.5 mM manganese(ii)-chloride tetrahydrate (MnCl_2_·4H_2_O, “Mn^2+^”) (Sigma Aldrich, USA). Buffer III consisted of buffer II with 1 μM tris-(2,2′-bipyridyl)-ruthenium(ii)-chloride hexahydrate (C_30_H_24_Cl_2_N_6_ Ru·6H_2_O, “RuPo”) (Sigma Aldrich, USA).

### 2.2 Lithography setup

MPL was performed with a customized lithography system (Workshop Of Photonics (WOP), Lithuania) using an ultra-short pulsed laser at 515 nm (CARBIDE, 1 MHz repetition rate, >290 fs pulse duration, Light Conversion) and a 3-axis stage (AEROTECH Nanopositioner, USA) for sample movement, as described previously.^92^ The same instrument was used for imaging in a confocal configuration. The scattered light was collected using an Avalanche PhotoDiode (APD) (PDM, Micro Photon Devices, Italy) operated with a custom-made software (*λ*_exc_ = 515 nm fs pulsed, *I* = 5 μW). MPL, imaging, and LAPAP were performed with an oil objective lens (63×, 1.4 NA, Zeiss, Germany).

### 2.3 Single molecule fluorescence microscopy (SMFM)

Fluorescence images were acquired using a modified Olympus IX81. A nanometre precision *XYZ* piezo stage (Physik Instrumente GmbH, Germany) on top of a mechanical stage with 1 × 1 cm range (JPK Instruments, Germany) was used for sample positioning. The sample was illuminated through a 60× magnification objective lens (60×, 1.42 NA, Olympus, Austria) and an additional 1.6× magnification tube lens with a 642 nm diode laser (Omicron, Germany). A dichroic filter (ZT405/488/561/640rpc, Chroma, Germany), an emission filter (446/523/600/677 nm BrightLine quad-band band-pass filter, Semrock, USA), and an additional emission filter (HQ 700/75 M, NC209774, Chroma, Germany) were used for imaging of the probes of the immobilized fluorophore–biomolecule conjugates (ATTO647N-cNP77 for Cy-LAPAP and ATTO655-SA for Cou-LAPAP and Rho-LAPAP). The fluorescence signal was detected using an Andor iXonEM+ 897 (back illuminated) EMCCD camera (16 μm pixel size) (Andor Technology, United Kingdom).^93^ 1500 images were recorded with 20 ms illumination time and 4.15 kW cm^−2^ excitation intensity at *λ*_exc_ = 640 nm.

### 2.4 Sample preparation

Two- and three-dimensional MPL acrylate scaffolds (photoresist described above) were prepared with an excitation intensity of 42 GW cm^−2^ at a 1 mm s^−1^ writing speed and developed by rinsing the sample with acetone (Roth, Germany) (air-dried). Surface passivation was achieved *via* incubation with 2% BSA solution in PBS for 1 h at 4 °C. The remaining BSA was washed out in 3 washing steps with PBS.

We prepared solutions of: 1 μM Cy3-NP77 oligonucleotide (Cy-LAPAP), 50 μM ATTO390-biotin (Cou-LAPAP) and 20 μM ATTO514-biotin (Rho-LAPAP) for LAPAP. Positioning of the laser's focal spot during LAPAP was achieved by detecting the backscattered signal from the interface between the acrylate scaffold and the surrounding solution using the confocal imaging system. LAPAP was performed at 515 nm excitation wavelength at 85 GW cm^−2^ excitation intensity, 3000 pulses per spot with a pulse length of 1 ps for Cy-LAPAP, 40 GW cm^−2^ excitation intensity, 10 000 pulses per spot with a pulse length of 1.5 ps for Cou-LAPAP, 530 kW cm^−2^ excitation intensity, 10 000 pulses per spot with a pulse length of 1.5 ps for Rho-LAPAP and 40 GW cm^−2^ excitation power, 5500 pulses per spot with a pulse length of 1.5 ps for Cy-LAPAP on 3D acrylate scaffolds. Each LAPAP setup was tested with buffers I, II and III. In case of buffers II and III, the sample was washed with a 1 mM EDTA in ddH_2_O once after the LAPAP procedure and washed thoroughly with PBS subsequently. Prior to imaging, the sample was incubated with 10 nM ATTO647N-cNP77 for 10 seconds (Cy-LAPAP) and 20 nM ATTO655-SA for 20 seconds (Cou-LAPAP, Rho-LAPAP) to reveal surface-bound oligonucleotides/biotins and was afterwards thoroughly washed with PBS.

### 2.5 Data analysis

To obtain the luminescence intensity from single molecules, a glass coverslip with sparsely distributed ATTO647N-cNP77 (corresponding to single emitter signal) or ATTO655-SA (multiple labels/SA) was imaged. The signal intensity was analysed using ImageJ;^94^ the mean single molecule intensity (SMI) was calculated (*n* = 40 single molecules analysed across 5 samples for each fluorophore). The SMI was applied to calculate the number of bound molecules (BM) on the MPL structures per pixel, by dividing the measured fluorescence intensity of the LAPAP spots on the structure by the SMI. The LAPAP-efficiency was calculated by dividing the number of fluorescent LAPAP spots along a 30 μm line by the total number of illuminated LAPAP spots (max. 15 LAPAP spots, separated by 2 μm). BM and LAPAP-efficiency were analysed on *n* = 45 images on *n* = 135 scaffolds.

The calculation of the position accuracy and the single molecule localization analysis was performed using 3DStormTools.^93,95^

### 2.6 Structural optimization of fluorophore/Mn^2+^ complexes

To identify potential binding sites for Mn^2+^ in Cy3, ATTO 390, and ATTO 514 fluorophores, we first constructed the fluorophore structures obtained from the retailer (see Chemicals section) in Avogadro.^96^ After a conjugate gradient potential energy minimization in Avogadro using universal force field (UFF),^97^ the resulting structures were optimized using density functional theory (DFT) with the B3LYP hybrid exchange correlation functional^98–100^ and the aug-cc-pVDZ basis set^101–103^ in implicit water at standard conditions (*ε*_r_ = 80.2^104^) using the integral equation formalism – polarizable continuum model (IEF-PCM) method.^105^ In IEF-PCM, UFF atomic radii were used for the calculation of molecular exclusion volumes. Cy3 and ATTO 390 were both modelled in their singlet ground state with zero net charge. ATTO 514 was modelled as a singlet with net charge +*e*. All DFT calculations were carried out using QCHEM v 5.41.^106^

In the second step, these optimized structures were used to calculate the electrostatic potential around the respective fluorophore in implicit water on a cubic grid with a maximum distance of 0.8 nm to any atom in the molecule. Using the chemical electrostatic potential – Boltzmann weighted (CHELP-BOW) method,^107^ we fitted atomic partial charges to the nuclear positions in the fluorophore that best reproduce the recorded electrostatic potential. By looking out for spatially clustered negative partial charges, we identified potential interaction sites for Mn^2+^ in the fluorophores.

To confirm our results from the partial charge fitting procedure, we placed Mn^2+^ directly in the proximity of the putative binding sites and performed another DFT potential energy optimization procedure with the same approach as described above. In these calculations, each Mn^2+^ carried a net charge of +2e and was assumed to be in the high spin state with five unpaired electrons. The high spin assumption can be justified by the spectroscopically identified ground state of the observed octahedral coordination of the [Mn(H_2_O)_6_]^2+^ complex,^108,109^ which is also observed during complexation with biomolecules.^78^

For Cy3 and ATTO 390, the investigated ion–fluorophore complexes were sextets with a net charge of +2*e*. On the other hand, ATTO 514 which binds two Mn^2+^, had a net charge of +5*e* and ten unpaired electrons. The optimized ion–fluorophore complexes were visualized and analysed using UCSF Chimera v 1.16.^110^

## 3 Results

**Fig. 1:**
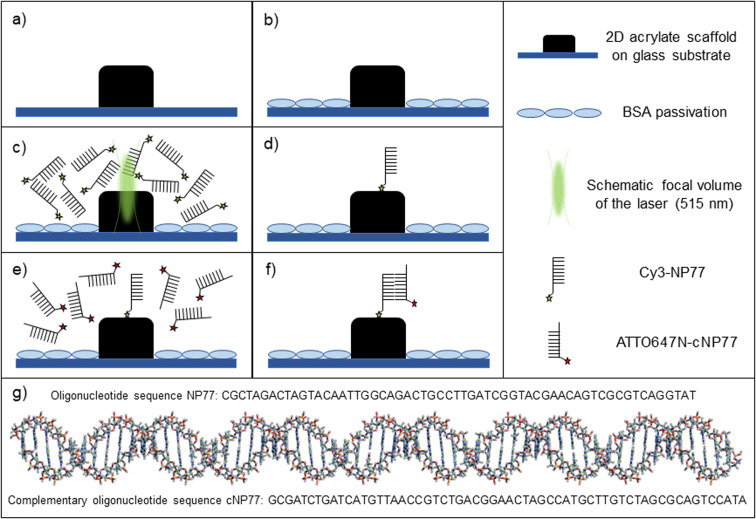
Schematic representation of the 2D acrylate scaffold functionalization *via* LAPAP at a single molecule level (smLAPAP). (a) A 2D acrylate scaffold (4 : 1 weight ratio Ormocomp : PEGDA, 1 wt% Irgacure 819) was prepared *via* MPL on glass substrate. (b) Passivation of the surrounding glass substrate with BSA. (c) Incubation with Cy3 conjugated to NP77- (Cy3-NP77) model oligonucleotide and photobleaching of the fluorophores in the focal spot of the laser (515 nm) (LAPAP). (d) After washing, only bleached fluorophores bound to the polymer-substrate remain on the scaffold. (e) Incubation with ATTO 647N labelled cNP77 (ATTO647N-cNP77) complementary oligonucleotide. (f) Immobilized oligonucleotide helix on the polymer surface. (g) Oligonucleotide helix composed of NP77 and cNP77. Sketches are not to drawn scale.

The newly developed buffer-systems have been tested for LAPAP using cyanine-, coumarin- and rhodamine-fluorophores conjugated to oligonucleotides or biotins. A sketch of the experiment is shown in Fig. 1a–f. 2D/3D MPL-structured acrylate scaffolds were used as a substrate for biomolecule immobilization. Prior to LAPAP, the glass substrate was passivated with a 2% solution of BSA next to 2D/3D MPL-acrylate scaffolds (ESI† Fig. S4). For single-molecule LAPAP (smLAPAP), the short-pulsed excitation beam was precisely positioned at the scaffold interface, and its duration, intensity and number of pulses were adapted to immobilize single biomolecules on the scaffold.

The impact of the new buffers on the dye-dependent smLAPAP efficiency was assessed *via* a quantitative single molecule fluorescence microscopy (SMFM) based analysis method. Precisely, the number of bound biomolecules (BM) was calculated by forming the ratio of the average fluorescence intensity within the illuminated LAPAP area and the mean single molecule intensity (SMI) (BM = mean intensity LAPAP area/SMI). The LAPAP-efficiency was calculated by dividing the number of fluorescent LAPAP spots by the total number of illuminated LAPAP spots. Fluorescence intensity calibration control experiments were independently performed to estimate the intensities of single ATTO647N-cNP77 and ATTO655-SA, which were used as probes to reveal the LAPAP immobilized biomolecules (see Materials and methods).

More specifically, for Cy-LAPAP, we immobilized a cyanine derivative (Cy3) conjugated to an NP77-model oligonucleotide (Cy3-NP77) on the acrylate scaffold. The immobilized oligonucleotides were detected by incubation with the fluorescently labelled complementary cNP77 oligonucleotide (ATTO647N-cNP77). For Cou-LAPAP we used a coumarin derivative (ATTO 390) conjugated to a biotin (ATTO390-biotin). For Rho-LAPAP we used a rhodamine derivative (ATTO 514) conjugated to a biotin (ATTO514-biotin). The immobilized biotins (Cou-LAPAP, Rho-LAPAP) were detected with ATTO 655 conjugated to a streptavidin (ATTO655-SA) and imaged by SMFM.

Ciuba *et al.*^78^ have previously shown that buffers containing paramagnetic transition metal ions (*e.g.* Mn^2+^) have a pronounced effect on photobleaching driven by enhanced ISC. In addition to Mn^2+^, we have investigated the effect of a co-initiator that promotes radical formation, *i.e.*, bleaching, of the fluorophore *via* enhanced singlet state oxygen formation. As a co-initiator, tris-(2,2′-bipyridyl)-ruthenium(ii)-chloride hexahydrate (RuPo) has been chosen, which is an efficient singlet oxygen generator^111–113^ with an absorption spectrum matching the LAPAP excitation wavelength^114^ (see ESI† Table S5 for presumed 1-photon or 2-photon absorption).

Therefore, we have developed two new buffer systems containing Mn^2+^ and Mn^2+^ + RuPo, respectively. Buffer I consists either of double-distilled water (Cy-LAPAP) or PBS (Cou-LAPAP, Rho-LAPAP). Buffer II consists of Mn^2+^ in buffer I. Buffer III consists of Mn^2+^ and RuPo in buffer I. All in all, we have tested three fluorophore classes with three buffers (see Materials and methods).

In a first step, the patterning capabilities were tested on 2D acrylate scaffolds. The scaffolds (10 × 100 × 2 μm bars with a periodicity of 10 μm) consisted of Ormocomp^89^ and poly(ethylene glycol)diacrylate (PEGDA, weight ratio 4 : 1) (see ESI† Fig. S1).

**Fig. 2:**
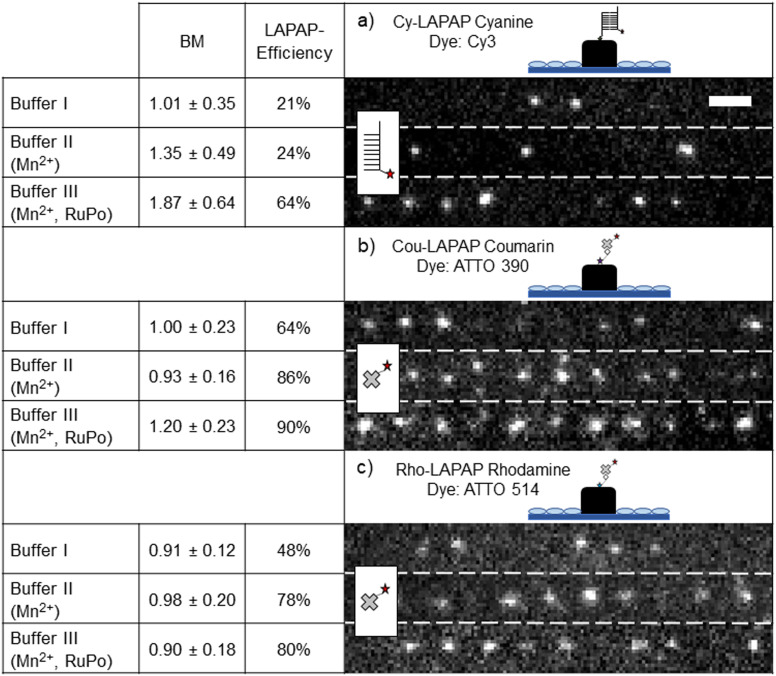
LAPAP on 2D scaffolds. 3 buffer systems (buffer I: neat water for Cy-LAPAP and PBS for Cou-LAPAP and Rho-LAPAP; buffer II: buffer I + Mn^2+^; buffer III: buffer I + Mn^2+^ + RuPo) with different fluorophore families were tested for LAPAP. Immobilized molecules were confirmed with ATTO647N-cNP77 and ATTO655-SA, respectively. (a) Cy-LAPAP cyanine dye: Cy3-NP77. With buffer I, 1.01 ± 0.35 BM at LAPAP-efficiency of 21% was observed. With buffer II, BM increases to 1.35 ± 0.49 with a LAPAP-efficiency of 24%. Buffer III yielded 1.87 ± 0.64 BM, and an increase of LAPAP-efficiency by three-fold (64%). (b) Cou-LAPAP coumarin dye: ATTO390-biotin. With buffer I, 1.00 ± 0.23 BM at LAPAP-efficiency of 64% was estimated. With buffer II, LAPAP-efficiency increased to 86% and 0.93 ± 0.16 BM. Buffer III yields 1.20 ± 0.23 BM with a LAPAP-efficiency of 90%. (c) Rho-LAPAP rhodamine dye: ATTO514-biotin. With buffer I, 0.91 ± 0.12 BM at LAPAP-efficiency of 48% was determined. With buffer II, LAPAP-efficiency increased to 78% and 0.98 ± 0.20 BM. Buffer III yielded similar results as buffer II. Scale bar for all fluorescence images 2 μm.

For precise positioning of the photobleaching laser beam on the polymer surface, the interface was determined by detecting the light scattered from the polymer/buffer interface. For that, our MPL lithography setup has been used in confocal imaging mode.^92^ Positioning in axial-direction (sub-100 nm) of the excitation point spread function (PSF) on the polymer surface, laser intensity and pulse duration were adjusted. For LAPAP optimization, parameters such as BM and LAPAP-efficiency were evaluated for each experiment (see Fig. 2).

For BM estimation, average SMIs of individual ATTO647N-cNP77 and ATTO655-SA probe molecules were determined prior to LAPAP experiments. The SMI of both probe molecules, sparsely distributed on a glass surface, was 357 ± 29 arb.u. and 292 ± 28 arb.u., respectively (see Materials and methods).

Fig. 2 shows the number of bound biomolecules (BM) and the LAPAP efficiencies for all dyes and buffer systems investigated. Hereby, LAPAP-efficiency refers to the percentage of fluorescent spots on the scaffold while BM refers to the average number of bound fluorophores on the scaffold, which do show fluorescence, estimated from the signals in a diffraction-limited LAPAP area (FWHM ∼330 nm, see ESI† Fig. S2). As shown in Fig. 2a, Cy-LAPAP with buffer I yielded 1.01 ± 0.35 BM and LAPAP-efficiency of 21% (*n*_images_ = 5, *n*_scaffold_ = 3/image histogram mode = 1). This means, that one out of five LAPAP-modified spots on the scaffold showed some fluorescence and, in those cases, the fluorescence intensity corresponded to that of one fluorophore, on average. Buffer II increased the BM to 1.35 ± 0.49 with 24% LAPAP efficiency (*n*_images_ = 4, *n*_scaffold_ = 3/image, histogram mode = 1). Buffer III yields 1.87 ± 0.64 BM, and LAPAP-efficiency is increased to 64% (*n*_images_ = 6, *n*_scaffold_ = 3/image, histogram mode = 2) (*t*_pulse_ = 1 ps, 3000 pulses per spot). For Cou-LAPAP with buffer I, we estimated on average 1.00 ± 0.23 BM and LAPAP-efficiency of 64% (*n*_images_ = 5, *n*_scaffold_ = 3/image histogram mode = 1). LAPAP-efficiency increased up to 86% (*n*_images_ = 5, *n*_scaffold_ = 3/image histogram mode = 1) with buffer II while BM remains at 0.96 ± 0.16. Buffer III yielded 1.20 ± 0.23 BM with a LAPAP-efficiency of 90% (*n*_images_ = 5, *n*_scaffold_ = 3/image histogram mode = 2) (*t*_pulse_ = 1.5 ps, 10 000 pulses per spot) (see Fig. 2b). For Rho-LAPAP with buffer I, we estimated 0.91 ± 0.12 BM at LAPAP-efficiency of 48% (*n*_images_ = 5, *n*_scaffold_ = 3/image histogram mode = 1). LAPAP-efficiency increased to 78% and BM at 0.98 ± 0.20 (*n*_images_ = 5, *n*_scaffold_ = 3/image histogram mode = 1) with buffer II. Buffer III yielded similar results to buffer II (*n*_images_ = 5, *n*_scaffold_ = 3/image histogram mode = 1) (*t*_pulse_ = 1.5 ps, 10 000 pulses per spot) (Fig. 2c). For a more comprehensive presentation of the BM within one LAPAP-modified spot, histograms are presented in ESI† Fig. S6. In total, 45 images and 135 scaffolds were analysed. ESI† Table S4 shows autofluorescence intensities of the acrylate scaffolds. In order to reveal the effect of RuPo on LAPAP, we tested Cy-LAPAP with buffer III without Mn^2+^ and observed no increase in BM and LAPAP-efficiency (see ESI† Fig. S5).

The highest LAPAP-efficiency was achieved for buffer III with Cou-LAPAP yielding up to 90% of functionalized diffraction limited LAPAP illumination areas (see Fig. 2b). The biggest increase in LAPAP-efficiency was observed for Cy-LAPAP. A three-fold increase from 21% to 64% LAPAP-efficiency was observed with buffer III (Fig. 2a).

**Fig. 3:**
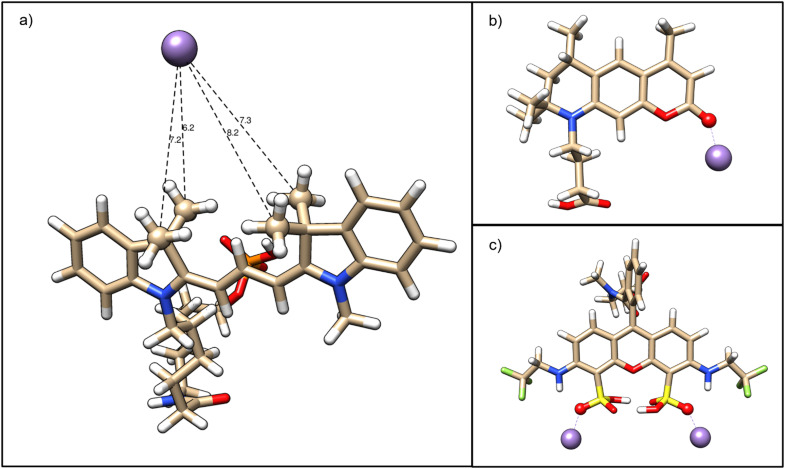
a) Optimized Cy3–Mn^2+^ complex with distances in Ångström, (b) optimal ATTO 390-Mn^2+^ and (c) ATTO 514-Mn^2+^ complexes, respectively, in implicit water (*ε*_r_ = 80.2). In Cy3, the four methyl groups attached to the fluorescent π system interact weakly with the Mn^2+^. In ATTO 390, the Mn^2+^ is attracted by the ketone oxygen shown in red, which is directly involved in the fluorophore's π system. In ATTO 514, two hydrogen sulphite groups can electrostatically bind Mn^2+^, effectively providing a 2 : 1 binding stoichiometry and potentially perturbing the peripheral, blue-coloured nitrogens of the π system. Mn^2+^ is shown in purple. Identified binding site atoms are shown in van-der-Waals representation.

To identify putative specific Mn^2+^ interaction sites with unconjugated fluorophores, quantum mechanical DFT calculations were performed with Cy3, ATTO 390 and ATTO 514 (see Materials and methods). As a first step, we calculated the optimal fluorophore structures in implicit water under standard conditions and fitted atomic partial charges to the minimum potential energy structures which reproduce the overall electrostatic potential of the fluorophore (see ESI† Table S1 for Cy3, ESI† Table S2 for ATTO 390 and ESI† Table S3 for ATTO 514). Indeed, our analysis for Cy3 revealed that the four methyl groups attached to the two pyrrole rings of the fluorescent π system carry an overall negative net charge of −0.26*e*. Therefore, the four methyl groups in Cy3 may provide a weak binding pocket for Mn^2+^. We tested this hypothesis *in silico* by directly calculating the optimized Cy3–Mn^2+^ complex with the Mn^2+^ ion placed in between the four methyl groups. The Mn^2+^ established a stable resting distance to the methyl carbons of 0.72 ± 0.04 nm, confirming our Cy3 binding site candidate (see Fig. 3a).

For ATTO 390 (see Fig. 3b), our approach identified a negatively charged furan ring oxygen (*q* = −0.57*e*) and an even more strongly negatively charged ketone oxygen (*q* = −0.68*e*) attached to the furan ring, which is directly connected to the π system. By placing our Mn^2+^ probe in the proximity of the two oxygens, we found that the ketone oxygen alone can electrostatically bind Mn^2+^.

Turning our attention to ATTO 514 (see Fig. 3c), we find that the hydrogen sulphite groups attached to the benzene rings of the π system can each bind independently a Mn^2+^. While the hydrogen sulphite groups structurally stabilize each other by virtue of two hydrogen bonds, the third hydrogen sulphite oxygen carries a charge of −0.41 e and can reliably bind a Mn^2+^. The Mn^2+^ is near an amino group, which is directly linked to the ring system and is therefore presumed to affect electron delocalization *via* the lone nitrogen electron.

In summary, our quantum-mechanical analyses have predicted binding sites for Mn^2+^ ions based on electrostatics, supporting the idea that direct ion–fluorophore interactions possibly affect LAPAP efficiency.

Overall, the experimental results for Mn^2+^-containing buffers are consistent with the results published by Ciuba *et al.*^78^ The calculations indicate that all fluorophores provide specific interaction sites for cations. These interaction sites are in proximity to the fluorophores' π-system and will most likely affect photobleaching and enhance LAPAP efficiency.

Our buffers allow efficient smLAPAP with 1.4 times reduced excitation intensity compared to standard buffers for tested fluorophore classes (for *e.g.*, Cy3-LAPAP: buffer I: 85 GW cm^−2^, with buffer III: 60 GW cm^−2^). At LAPAP excitation intensity settings greater than 90 GW cm^−2^ (*t*_pulse_ = 1 ps, 250 pulses per spot) the damage threshold is exceeded, and upon photo damage, the illuminated areas exhibit an unbleachable autofluorescence. In comparison, the “true” LAPAP spots were only visible after incubation and showed a stepwise photobleaching behaviour (see ESI† Fig. S3). The combined decrease of photodamage likelihood and excitation intensity for enhanced LAPAP-efficiency in buffer III will allow for a precise adjustment of the required pulse number for consistently bound single molecules. Additionally, a dependence of the damage threshold on the pulse duration and number of pulses was observed. Polymer damage for 1 ps pulses occurred at four times smaller number of pulses compared to 1.5 ps pulses at constant laser output power. To ensure reproducible smLAPAP results for Cou-LAPAP and Rho-LAPAP, the pulse duration was kept at 1.5 ps.

**Fig. 4:**
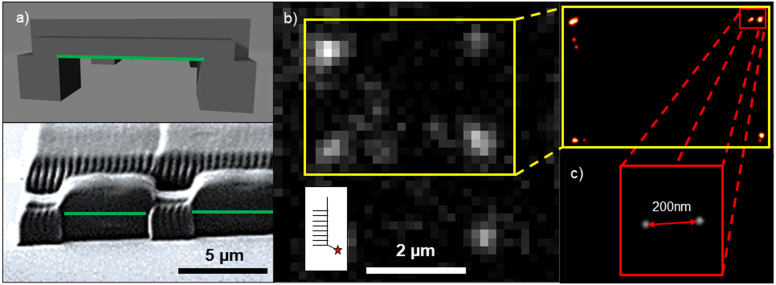
LAPAP on a 3D scaffold (a) model sketch and SEM image of 3D MPL acrylate scaffold. The laser was focused on the bottom of the hanging horizontal bars (green lines). (b) Fluorescence microscopy image of fluorescent spots on 3D MPL acrylate scaffold. Cy-LAPAP with buffer III was used. An average BM of 1.45 ± 0.36 was calculated. (c) Localized positions of emitters in yellow insert in (b), in the red insert two bound ATTO647N-cNP77 separated by ∼200 nm (average position accuracy PA: 24 ± 5 nm) within one diffraction limited spot are resolved. Gray value scaled to maximum.

We show promising LAPAP results for 3D MPL-printed acrylate scaffolds. Here, the 3D scaffolds consist of 5 × 100 × 2 μm horizontal grids on top of 5 × 5 × 5 μm pillars. Fig. 4a presents a model and a scanning electron microscopy (SEM) image of the scaffold. Here, we focused the laser beam on the overhanging horizontal bars (green lines in Fig. 4a). The best results for smLAPAP were obtained for *t*_pulse_ = 1.5 ps and 5500 pulses per spot, as shown in Fig. 4b. The best LAPAP-results were found for Cy-LAPAP and buffer III with an average value of 1.45 ± 0.36 BM (*n* = 30 spots on 3 technical replicates). No immobilized biomolecules were detected for LAPAP excitation intensities below the damage threshold using buffer I and buffer II and shorter pulse duration.

Single molecule localization microscopy (SMLM) was applied to confirm the results on single molecule analysis of the BM.^115,116^ SMLM allows the determination of the emitter position at a sub-50 nm scale, based on the detected PSF of the emitter. To avoid additional aberrations introduced by the polymers, the biomolecules were patterned only on the overhanging parts of the structure.

Fig. 4b shows fluorescence signals of ATTO647N-cNP77 complementing Cy3-NP77 immobilized *via* LAPAP on a 3D MPL scaffold Fig. 4c illustrates the localized emitters of the 4 spots in the yellow insert in Fig. 4b. In the red insert two ATTO647N-cNP77 are presented. The emitters are separated by ∼200 nm within the LAPAP spot. Each emitter has been localized with an average position accuracy (PA) of 24 ± 5 nm. Single molecule intensity analysis of the same spot confirms this assumption (intensity of spot = 702 arb.u. divided by SMI = 357 ± 29 arb.u. = 1.97 BM).

## Conclusions

In summary, we demonstrated that our newly developed buffer systems significantly improve the LAPAP-efficiency and allow for reduced painting power, enabling smLAPAP on MPL structured 2D and 3D acrylate scaffolds. We showed that our buffer systems improve LAPAP efficiency for Cy3, ATTO 514 and ATTO 390.

Increased LAPAP efficiency can be attributed to specific electrostatic Mn^2+^–fluorophore interactions as previously argued by Ciuba *et al.*,^78^ which we characterized in quantum mechanical DFT calculations. More precisely, the calculations predict a rather weak binding pocket for Mn^2+^ in Cy3, where LAPAP efficiency increases only slightly. For Cou-LAPAP and Rho-LAPAP, where the Mn^2+^ binding is stronger, the LAPAP efficiency increased from 64% to 86% and from 48% to 78%, respectively. The oligonucleotide-, biotin-modifications have not been considered in the calculations. Additional enhancement of the LAPAP efficiency has always been observed for RuPo-containing buffers. The presented experimental results clearly show the positive influence of paramagnetic transition metal ions and ROS on LAPAP efficiency and could expand its application range as a surface modification tool. Further investigations, in particular spectroscopic and computational studies, will be needed to thoroughly characterize the effects of ligand binding on the photobleaching reaction as well as ISC rates and to further refine the LAPAP-mechanism.

Our results pave the way for future LAPAP applications with low power light sources, including but not limited to surface modifications for biotechnological application for *e.g.*, 3D cell cultures or *in vitro* assays as well as molecular sensors which require local modifications.

## Author contributions

J. J. and C. N. designed the experiments. C. N. performed the experiments and analysed the data. H. K. performed the quantum mechanical calculations. G. G. distributed 2D and 3D scaffolds. D. S. and T. K. helped interpret and analyse the data. C. N. H. K., D. S. and J. J. wrote the manuscript. All authors reviewed and approved the final manuscript.

## Conflicts of interest

There are no conflicts to declare.

## Supplementary Material

LF-001-D3LF00125C-s001
