# Supplementary material for: New buffer systems for photopainting of single biomolecules
Source: RSC Appl Interfaces. 2023 Oct 7;1(1):110–21. doi: 10.1039/d3lf00125c (PMC10805099; doi:10.1039/d3lf00125c)
Supplement: LF-001-D3LF00125C-s001 [file LF-001-D3LF00125C-s001.pdf]

Electronic Supplementary Material (ESI) for RSC Applied Interfaces.
This journal is © The Royal Society of Chemistry 2023

Photopainting of Single Molecules Using New Buffer Systems

Supporting Information

Christoph Naderer[1], Heinrich Krobath[2], Dmitry Sivun[1], Georgii Gvindzhiliia[3], Thomas A. Klar,[3] and Jaroslaw Jacak,[1]*

[1] School of Medical Engineering and Applied Social Sciences, University of Applied Sciences Upper Austria, Garnisonstraße. 21, 4020 Linz, Austria

[2] Institute of Theoretical Physics, Johannes Kepler University Linz, Altenberger Straße 69, 4040 Linz, Austria

[3] Institute of Applied Physics, Johannes Kepler University Linz, Altenberger Straße 69, 4040 Linz, Austria

Chemical element	x-coordinate (Å)	y-coordinate (Å)	z-coordinate (Å)	Partial charge (e)
C	-3.719948	-3.618961	-1.551950	0.670877
O	-2.976587	-4.540301	-1.915643	-0.668615
C	-3.218315	-1.190279	-2.204373	0.347197
C	-3.180180	-2.267201	-1.099222	-0.451084
H	-3.756746	-1.910277	-0.234036	0.116075
H	-2.146098	-2.427863	-0.770637	0.116075
C	-2.376954	-1.542406	-3.438341	-0.275552
H	-2.862925	-0.251983	-1.756050	-0.038144
H	-4.260319	-1.013970	-2.511119	-0.038144
C	-2.449723	-0.506416	-4.569304	-0.080068

H	-2.712679	-2.507392	-3.841573	0.096138
H	-1.327166	-1.685355	-3.137068	0.096138
C	-1.821832	0.843323	-4.191438	-0.093523
H	-3.496594	-0.347594	-4.865808	0.075253
H	-1.927864	-0.901352	-5.451703	0.075253
N	-1.797490	1.787075	-5.313365	-0.165014
H	-0.795743	0.710849	-3.832439	0.122511
H	-2.375038	1.330108	-3.382308	0.122511
C	-2.860472	2.681540	-5.573131	0.157084
C	-0.806409	1.909784	-6.229499	0.135943
C	-2.544641	3.443705	-6.700541	0.033382
C	-1.181311	3.024130	-7.224609	0.397329
**C**	**-1.305075**	**2.490481**	**-8.670616**	**-0.447349**
**H**	**-1.975924**	**1.624537**	**-8.712936**	**0.125402**
**H**	**-0.333814**	**2.196142**	**-9.082558**	**0.125402**
**H**	**-1.717754**	**3.278793**	**-9.311359**	**0.125402**
**C**	**-0.195045**	**4.213342**	**-7.144741**	**-0.459114**
**H**	**-0.571410**	**5.035806**	**-7.764533**	**0.128878**
**H**	**0.799702**	**3.940764**	**-7.512342**	**0.128878**
**H**	**-0.096433**	**4.574528**	**-6.114337**	**0.128878**
C	-4.053743	2.843671	-4.871039	-0.159813
C	-4.943356	3.821097	-5.337286	-0.183730
C	-4.641373	4.596916	-6.463171	-0.117842
C	-3.433276	4.411135	-7.154552	-0.267429
H	-5.886165	3.974559	-4.812749	0.162064
H	-4.304465	2.245966	-3.997492	0.139136
H	-3.207105	5.018908	-8.030642	0.186871
C	0.337604	1.105798	-6.202347	-0.257345
C	1.424844	1.177735	-7.076524	-0.126325
C	2.533716	0.329666	-6.982392	-0.180833
C	3.653974	0.336037	-7.817061	0.003218
N	4.669814	-0.544385	-7.646829	0.064037
C	3.976560	1.248636	-9.013156	0.366089
C	5.329938	0.705454	-9.441361	0.002966
C	5.693259	-0.353056	-8.602929	0.179899
**C**	**2.948432**	**1.080906**	**-10.156188**	**-0.423277**

**H**	**1.963290**	**1.467131**	**-9.874129**	**0.122970**
**H**	**2.839613**	**0.027079**	**-10.438029**	**0.122970**
**H**	**3.291265**	**1.638720**	**-11.035921**	**0.122970**
**C**	**4.104438**	**2.729561**	**-8.585537**	**-0.465777**
**H**	**3.139370**	**3.150331**	**-8.282905**	**0.134177**
**H**	**4.479024**	**3.318989**	**-9.431095**	**0.134177**
**H**	**4.807712**	**2.839762**	**-7.751658**	**0.134177**
C	4.705424	-1.572915	-6.612925	-0.337559
C	6.173577	1.092224	-10.475908	-0.228404
C	7.384257	0.404136	-10.655729	-0.165669
C	7.732793	-0.653122	-9.806959	-0.103467
C	6.889135	-1.051849	-8.761300	-0.291835
H	5.617464	-2.159807	-6.724698	0.154303
H	3.840114	-2.239098	-6.713548	0.154303
H	4.699106	-1.112947	-5.617073	0.154303
H	-5.350456	5.349790	-6.806600	0.144560
H	5.909364	1.912839	-11.142748	0.183179
H	8.057578	0.695490	-11.461440	0.150997
H	8.675335	-1.179043	-9.957561	0.147431
H	7.176832	-1.875928	-8.112552	0.189027
N	-5.071180	-3.737141	-1.560782	-0.406468
C	-5.774249	-4.947689	-1.979330	-0.101607
H	-5.621445	-2.977384	-1.184020	0.272247
C	-7.253224	-4.657310	-2.229212	0.191690
H	-5.291280	-5.326363	-2.889442	0.075507
H	-5.665747	-5.728752	-1.209605	0.075507
C	-8.044161	-5.909228	-2.622352	0.152007
H	-7.342967	-3.896638	-3.020277	-0.032993
H	-7.695305	-4.220966	-1.318397	-0.032993
C	-9.535607	-5.622861	-2.835803	0.022553
H	-7.607116	-6.339429	-3.536435	-0.046840
H	-7.933740	-6.673074	-1.835382	-0.046840
C	-10.377596	-6.867374	-3.152285	-0.157136
H	-9.657525	-4.884906	-3.642452	-0.015528
H	-9.938218	-5.155888	-1.923446	-0.015527
C	-10.106925	-7.511534	-4.507983	-0.068681

H	-11.445866	-6.607155	-3.105212	0.070612
H	-10.206529	-7.634758	-2.379870	0.070612
O	-10.471949	-6.587628	-5.547028	-0.349723
H	-10.703380	-8.431245	-4.609746	0.088375
H	-9.045195	-7.784054	-4.615309	0.088375
P	-10.358104	-7.131543	-7.131763	1.055435
O	-8.889910	-7.228170	-7.545152	-0.766778
O	-10.976611	-5.758697	-7.861149	-0.677437
O	-11.327079	-8.302004	-7.333926	-0.809326
H	-11.942430	-5.800300	-7.849003	0.415925
H	0.393149	0.342386	-5.427856	0.177752
H	1.409821	1.922555	-7.865314	0.169674
H	2.514857	-0.406634	-6.179607	0.149089

Suppl. Table 1 *Atomic partial charges of the optimized neutral Cy3 singlet ground state in implicit water using the CHELP-BOW method. The first column denotes the chemical element, columns 2-4 are Cartesian (x,y,z) coordinates in Å, the fifth column denotes the atomic partial charge in units of the elementary charge. The four methyl groups that jointly bind Mn^{2+} are highlighted in red (group 1), green (group 2), blue (group 3) and orange (group 4). All groups provide a net charge of -0.26e.*

Chemical element	x-coordinate (Å)	y-coordinate (Å)	z-coordinate (Å)	Partial charge (e)
C	-5.958643	3.459513	0.266910	-0.522513
C	-6.228420	1.947867	0.218880	0.723863
N	-5.035452	1.267184	-0.366420	-0.559491
C	-3.764684	1.799668	-0.258188	0.496174
C	-3.520055	3.062358	0.387944	-0.366951
C	-4.675573	3.830187	1.007711	0.587497
H	-6.820127	3.945699	0.742930	0.123161
H	-5.900224	3.840776	-0.764109	0.123161
C	-4.485426	5.353255	1.029416	-0.519407
H	-4.778651	3.505127	2.055683	-0.056649
H	-4.294910	5.743942	0.019703	0.122413
H	-3.656019	5.659833	1.678507	0.122413
H	-5.394427	5.833353	1.416253	0.122413
C	-7.464232	1.734786	-0.675822	-0.598093

H	-7.288193	2.115273	-1.689834	0.154174
H	-8.308569	2.290135	-0.248128	0.154174
H	-7.770019	0.684199	-0.737806	0.154174
C	-6.511033	1.388674	1.629505	-0.551445
H	-5.637437	1.490889	2.283289	0.138357
H	-6.777327	0.324805	1.588947	0.138357
H	-7.353702	1.923453	2.087537	0.138357
C	-5.193174	-0.123891	-0.793612	0.124791
C	-5.294273	-0.315401	-2.317496	0.040466
H	-6.096486	-0.526951	-0.325930	0.057567
H	-4.362137	-0.721413	-0.394983	0.057567
C	-5.405004	-1.812824	-2.672459	-0.542874
H	-4.415049	0.112050	-2.815421	0.050271
H	-6.169133	0.229154	-2.692131	0.050271
C	-5.481395	-2.012080	-4.172020	0.818514
H	-6.285030	-2.253337	-2.181601	0.156614
H	-4.516058	-2.347540	-2.317789	0.156614
O	-4.518468	-2.185535	-4.888171	-0.575780
O	-6.714979	-1.949286	-4.739091	-0.627477
H	-7.403306	-1.833093	-4.067675	0.454778
C	-2.223459	3.546840	0.447157	0.039086
C	-1.106827	2.862138	-0.082499	-0.598490
C	-1.372397	1.628395	-0.702158	0.730915
C	-2.654443	1.105199	-0.795934	-0.654640
H	-2.049007	4.502664	0.934179	0.111828
H	-2.761830	0.159469	-1.315030	0.261073
**O**	**-0.364259**	**0.880592**	**-1.261433**	**-0.569729**
C	0.958911	1.303032	-1.245397	0.992669
C	0.253842	3.336037	-0.038498	0.662841
C	1.234547	2.565508	-0.605771	-0.822550
C	0.586108	4.649722	0.613861	-0.532356
H	0.057114	5.476786	0.118776	0.156617
H	1.662031	4.849058	0.567538	0.156617
H	0.275497	4.653172	1.668503	0.156617
**O**	**1.775153**	**0.562177**	**-1.777355**	**-0.680066**
H	2.277191	2.876787	-0.601449	0.244109

Suppl. Table 2 *Atomic partial charges of the optimized neutral ATTO 390 singlet ground state in implicit water using the CHELP-BOW method. The first column denotes the chemical element, columns 2-4 are Cartesian (x,y,z) coordinates in Å, the fifth column denotes the atomic partial charge in units of the elementary charge. The ketone oxygen binding* Mn^{2+} *is highlighted in red bold, the proximal furan oxygen in green bold.*

Chemical element	x-coordinate (Å)	y-coordinate (Å)	z-coordinate (Å)	Partial charge (e)
C	-4.435303	2.015384	-2.170356	0.515854
F	-5.351347	2.721960	-2.866452	-0.167206
F	-4.855291	1.949537	-0.885597	-0.173201
F	-4.439213	0.746145	-2.653757	-0.163160
C	-3.065943	2.663484	-2.298348	-0.410773
N	-2.064107	1.820099	-1.670720	-0.133820
H	-3.131614	3.639293	-1.806912	0.224978
H	-2.866074	2.813973	-3.368968	0.224978
C	-0.841983	2.224813	-1.287397	0.288678
H	-2.245111	0.818112	-1.624290	0.274151
C	-0.451304	3.598582	-1.430979	-0.164507
C	0.777834	4.045759	-1.040226	-0.247910
C	1.751503	3.170723	-0.472739	0.289780
C	0.119146	1.328048	-0.712093	-0.248217
C	1.367809	1.815541	-0.316454	0.161744
H	-1.149735	4.304518	-1.868832	0.176726
H	1.021101	5.098254	-1.157714	0.205286
S	-0.208419	-0.425558	-0.460335	0.802752
**O**	**-1.523876**	**-0.758374**	**-1.050668**	**-0.409749**
O	0.894141	-1.174956	-1.370522	-0.432136
O	0.009987	-0.783961	0.974283	-0.454736
H	1.706721	-1.405330	-0.831777	0.406432
C	3.040860	3.576776	-0.057631	-0.304982
C	3.905099	2.618994	0.520923	0.357664
O	2.224602	0.923621	0.238586	-0.086773
C	3.463114	1.281389	0.660667	0.088256

C	5.233783	2.900867	0.957309	-0.356449
C	6.028487	1.935837	1.505704	-0.116872
C	5.579735	0.581972	1.669556	0.278540
C	4.250744	0.274170	1.226184	-0.195617
H	5.619749	3.910304	0.844606	0.253686
H	7.027849	2.203346	1.834135	0.170203
S	3.536167	-1.372819	1.354695	0.797217
**O**	**4.481696**	**-2.252089**	**2.077303**	**-0.407173**
O	2.263253	-1.184042	2.330418	-0.434198
O	3.064485	-1.831903	0.012478	-0.456013
H	1.427780	-1.025399	1.802094	0.403304
N	6.393419	-0.339201	2.210780	-0.156740
C	7.733084	-0.070343	2.704813	-0.383693
C	8.463359	-1.381156	2.945542	0.544744
H	7.718505	0.481598	3.655197	0.213968
H	8.322482	0.493684	1.973349	0.213968
F	7.764766	-2.200605	3.773627	-0.170992
F	9.664391	-1.147639	3.515456	-0.175397
F	8.677245	-2.069558	1.800510	-0.179667
H	5.993321	-1.261785	2.374591	0.269607
C	3.457710	5.005272	-0.147986	0.183810
C	3.512633	5.755610	1.035319	-0.191355
C	3.789971	5.610950	-1.379749	0.065332
C	3.913830	7.093852	1.007525	-0.149128
C	4.213906	6.946210	-1.387063	-0.159535
C	4.278487	7.685247	-0.203330	-0.126324
H	3.236401	5.291428	1.981240	0.170486
H	3.951802	7.665053	1.934240	0.168017
H	4.617266	8.720246	-0.228627	0.161192
H	4.517867	7.409272	-2.323967	0.142489
C	3.853636	4.747145	-2.616270	0.308073
O	4.442127	3.657141	-2.531266	-0.515083
N	3.284082	5.175266	-3.772462	-0.029990
C	3.477807	4.364609	-4.982313	-0.068404
C	2.474528	6.383832	-3.928779	-0.439348
H	1.557424	6.125994	-4.472873	0.167697

H	3.011390	7.155508	-4.497880	0.167697
H	2.195253	6.795520	-2.958184	0.167697
C	4.713250	4.798673	-5.781955	0.319387
H	2.570156	4.465201	-5.591110	0.076136
H	3.572167	3.318266	-4.675281	0.076136
C	4.861010	3.972048	-7.059527	-0.439864
H	5.607665	4.679105	-5.158007	-0.029942
H	4.638168	5.862661	-6.040616	-0.029942
C	6.064545	4.361805	-7.894796	0.845578
H	3.965396	4.072879	-7.692844	0.109128
H	4.947264	2.900275	-6.820491	0.109128
O	6.849147	5.243259	-7.621765	-0.598570
O	6.251891	3.648474	-9.036751	-0.636723
H	5.563118	2.975763	-9.147554	0.443740

Suppl. Table 3 *Atomic partial charges of the optimized positively charged ATTO 514 singlet ground state in implicit water using the CHELP-BOW method. The first column denotes the chemical element, columns 2-4 are Cartesian (x,y,z) coordinates in Å, the fifth column denotes the atomic partial charge in units of the elementary charge. The hydrogen sulphite groups are highlighted in red and green, respectively.* Mn^{2+}*-binding hydrogen sulphite oxygens are shown in bold.*

	Background acrylate scaffold	Background passivated glass substrate
Cy-LAPAP	194 ± 13 arb.u.	185 ± 16 arb.u.
Cou-LAPAP	170 ± 21 arb.u.	155 ± 11 arb.u.
Rho-LAPAP	155 ± 12 arb.u.	154 ± 10 arb.u.
Cy-LAPAP on 3D scaffold	250 ± 36 arb.u.	

Suppl. Table 4 *Background intensity for Cy-LAPAP, Cou-LAPAP, Rho-LAPAP and Cy-LAPAP on 3D acrylate scaffolds on acrylate scaffolds and the surrounding passivated glass substrate. For Cy-LAPAP on 3D scaffolds, the glass substrate was not analysed.*

Excitation wavelength 515nm	1-photon absorption	2-photon absorption
Cy3	X	
ATTO 390		X
ATTO 514	X	
RuPo		X

Suppl. Table 5 *Presumed 1-photon and 2-photon absorption for light of 515 nm wavelength.*

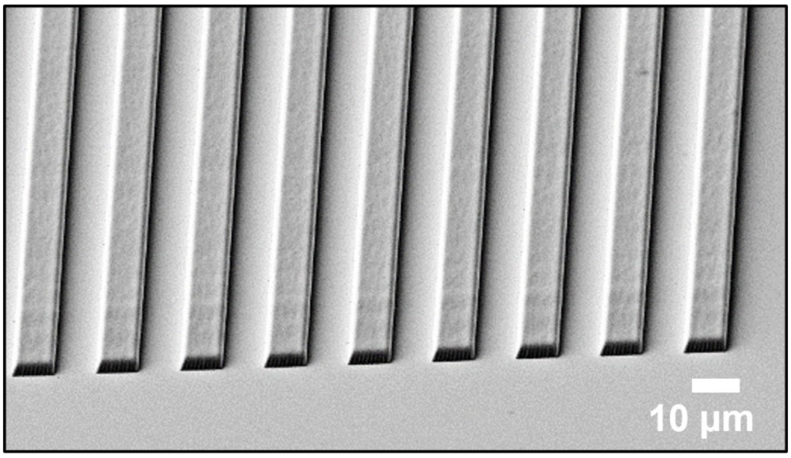

Suppl. Figure 1 *(c) SEM image of 2D MPL acrylate scaffold. of 10 x 100 x 2 µm bars with a periodicity of 10 µm.*

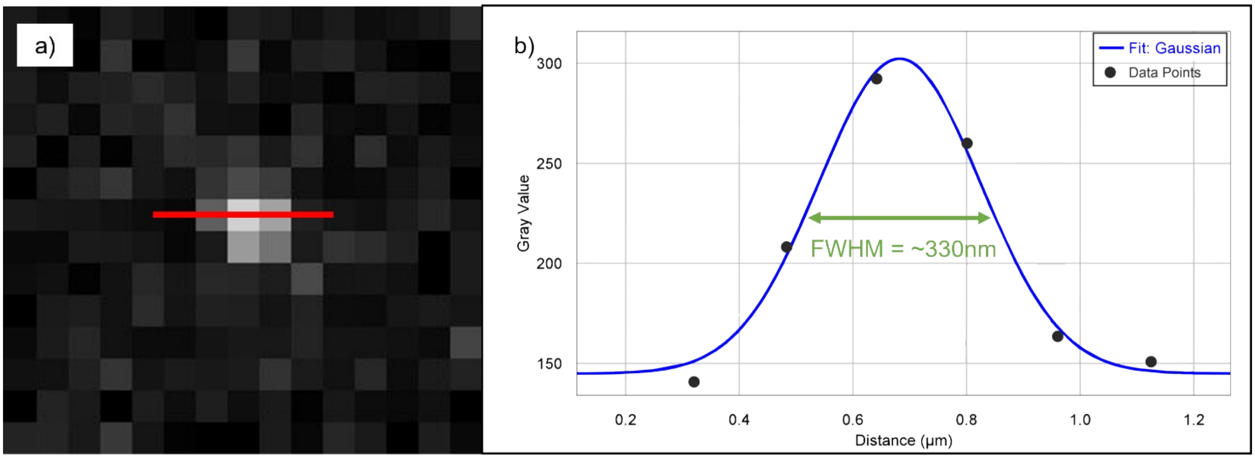

Suppl. Figure 2 *Fluorescence microscopy image and Gaussian fit for a single immobilized ATTO647N-cNP77. (λex = 640 nm, till = 20 ms, I = 4.15 kW/cm²). (a) Fluorescence microscopy image of a single ATTO647N-cNP77 bound to Cy3-NP77 immobilized via LAPAP on a 2D acrylate*

scaffold. The red line indicates the cross-section for the gaussian fit. (b) Experimental data and Gaussian fit for the fluorescence signal. A full width at half maximum (FWHM) of ~330 nm was calculated.

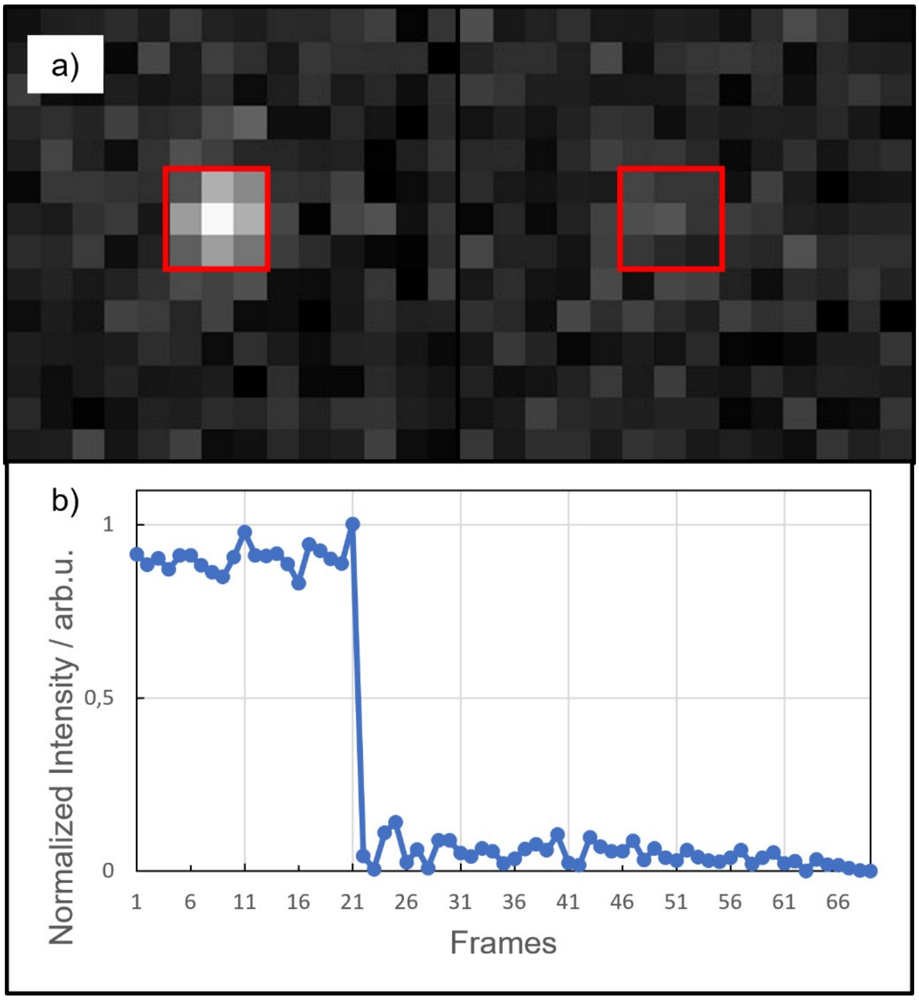

Suppl. Figure 3 *Fluorescence microscopy image and stepwise photobleaching behaviour of a single ATTO647N-cNP77. (λex = 640 nm, till = 20 ms, I = 4.15 kW/cm²). (a) Fluorescence microscopy image of a single ATTO647N-cNP77 bound to Cy3-NP77 immobilized via LAPAP on a 2D acrylate structure. The left image shows the fluorophore at the beginning of the sequence. The right image shows the same area at the end of the imaging sequence. The red square indicates the area for intensity stepwise photobleaching analysis. (b) Stepwise photobleaching behaviour over the first 70 frames of a 1000 frames sequence of a single ATTO647N-cNP77.*

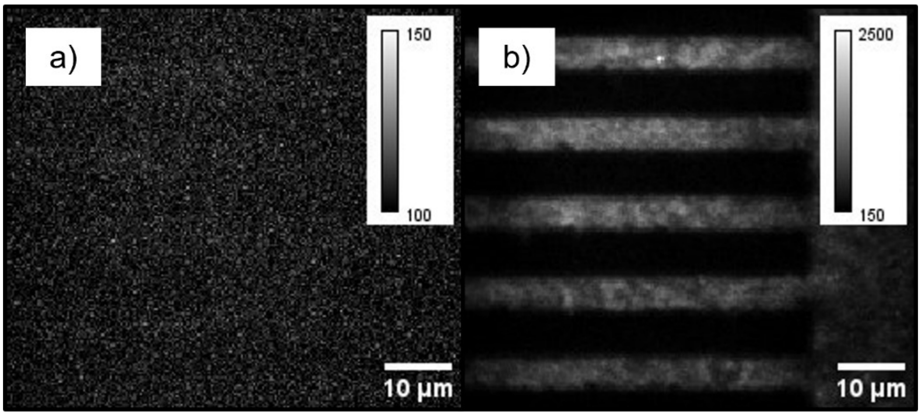

Suppl. Figure 4 *Fluorescence microscopy image of 2D acrylate structures before and after incubation with ATTO488-BSA conjugate. (λ_{ex} = 488 nm, t_{ill} = 20 ms, I = 3.78 kW/cm²). (a) Fluorescence microscopy image of 2D acrylate structure in PBS. (b) Fluorescence microscopy image of the same structure after 5 min incubation with ATTO488-BSA conjugate. The surrounding glass substrate shows strong fluorescence due to the bound BSA. The acrylate structure shows no unspecific binding of the protein.*

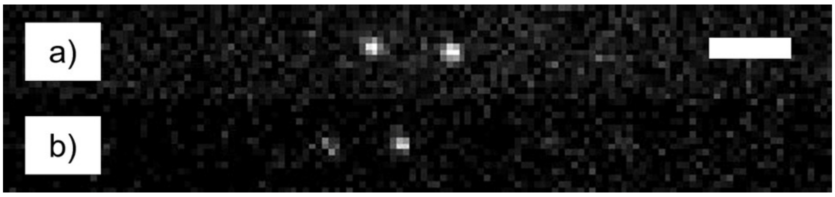

Suppl. Figure 5 *Fluorescence microscopy images of fluorescent dots on 2D MPL acrylate structure. (a) Cy-LAPAP with buffer I for reference (further description see Figure 3). (b) Cy-LAPAP with buffer III without Manganese. Without the addition of Mn^{2+}, no improvement was observed. Scale bar 2 µm.*

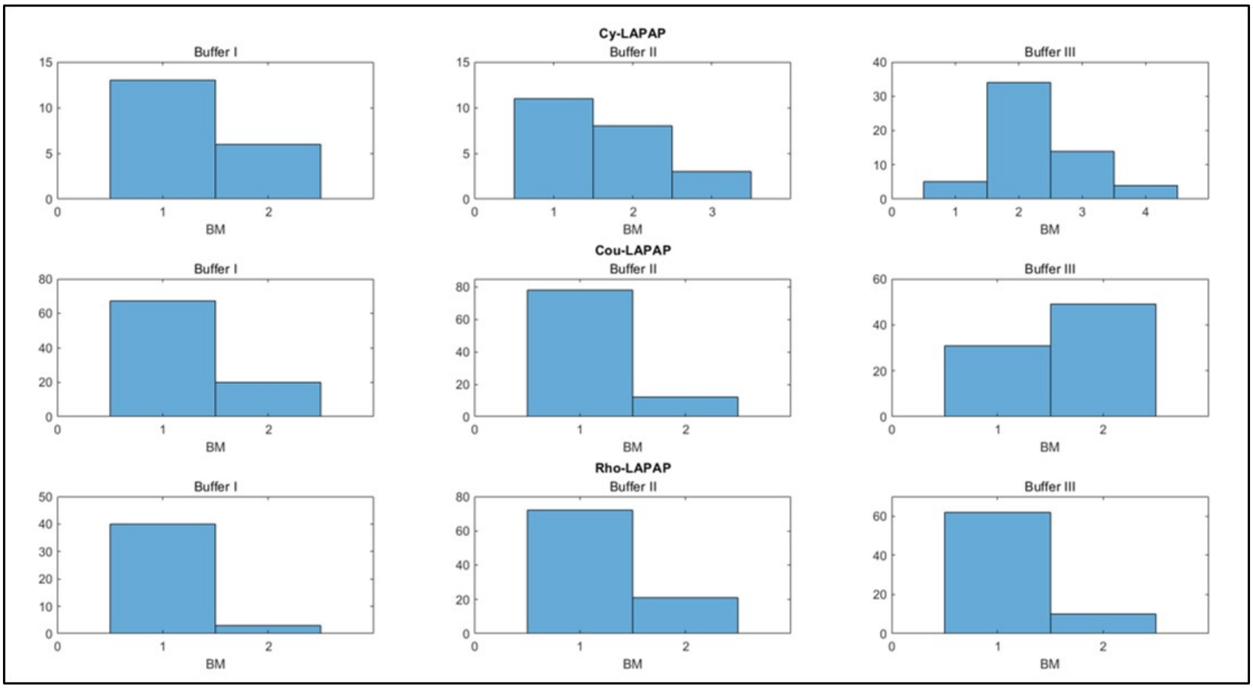

Suppl. Figure 6 *Histograms of number of bound biomolecules (BM) with Cy-LAPAP, Cou-LAPAP and Rho-LAPAP on 2D acrylate scaffolds corresponding to the calculated BM.*
